# Genotype-negative multiple endocrine neoplasia type 1 with prolactinoma, hyperparathyroidism, and subclinical Cushing’s syndrome accompanied by hyperglycemia: a case report

**DOI:** 10.3389/fendo.2024.1498991

**Published:** 2024-12-12

**Authors:** Haremaru Kubo, Ryota Wada, Naohiro Sekikawa, Yasuhisa Nomura, Mutsuo Yamada, Minoru Inoue, Naoki Hattori, Yuto Yamazaki, Kazuhiro Sugimoto

**Affiliations:** ^1^ Diabetes Center, Ohta Nishinouchi Hospital, Koriyama, Fukushima, Japan; ^2^ Department of Obstetrics and Gynecology, Ohta Nishinouchi Hospital, Koriyama, Fukushima, Japan; ^3^ Department of Surgery, Ohta Nishinouchi Hospital, Koriyama, Fukushima, Japan; ^4^ Department of General Internal Medicine, Ohta Nishinouchi Hospital, Koriyama, Fukushima, Japan; ^5^ Department of Orthopedic Surgery, Kansai Medical University, Osaka, Japan; ^6^ Department of Pathology, Tohoku University Graduate School of Medicine, Sendai, Japan

**Keywords:** multiple endocrine neoplasia type 1, prolactinoma, primary hyperparathyroidism, subclinical Cushing’s syndrome, hyperglycemia

## Abstract

**Background:**

Multiple endocrine neoplasia type 1 (MEN1) is a rare autosomal dominant disorder, accompanied by multiple endocrine neoplasms of the parathyroid, pancreas, pituitary, and other neoplasms in the adrenal glands. However, in some cases, patients clinically diagnosed with MEN1 may be genotype-negative.

**Case presentation:**

A 56-year-old female was diagnosed with MEN1 based on a macroprolactinoma (19 mm in diameter), primary hyperparathyroidism, and a cortisol-producing adrenal adenoma, without a family history. At first appearance, she had a hemoglobin A1c of 12.0% and a fasting plasma glucose level of 16.3 mmol/L (294 mg/dL). She complained of headaches and had a history of prolactinoma at 28 years of age, with concomitant elevated prolactin 1102.0 μg/L (ng/mL). Insulin therapy was initiated for glucose management following the administration of an oral hypoglycemic agent. Additionally, cabergoline was initiated for due to the prolactinoma, resulting in a normalized prolactin level. Thereafter, medication for diabetes could be withdrawn. Subsequently, surgery was performed for primary hyperparathyroidism and the cortisol-producing adrenal adenoma, which was consistent with the preoperative diagnosis. Additionally, a thyroid tumor resected with primary hyperparathyroidism revealed to be invasive papillary thyroid carcinoma (PTC). Target gene testing revealed a negative genotype for *MEN1* gene, with only one common polymorphism that was non-pathogenic.

**Conclusions:**

Genotype-negative MEN1 typically has a favorable clinical course without a third primary MEN1 manifestation. However, the present case had a symptomatic macroprolactinoma with an apparent elevated glucose level and three manifestations of tumors (pituitary, parathyroid, and adrenal) with invasive PTC, and a delayed diagnosis could have caused crucial deterioration. Clinicians should pay attention to the clinical features of MEN1 including glucose intolerance. In such cases, the treatment of endocrine disorders can lead to the normalization of the glucose level.

## 1 Introduction

Multiple endocrine neoplasia type 1 (MEN1) is a rare autosomal dominant disease accompanied by multiple endocrine neoplasms in the parathyroid, pancreas, and pituitary, and other neoplasms in the adrenal glands (1). The diagnosis is established in a proband with two or more endocrine neoplasms, including parathyroid, pituitary, and gastroenteropancreatic (GEP)-neuroendocrine tumors (NET), or one of three endocrine neoplasms (parathyroid, pituitary, or GEP-NET) in a first-degree relative with MEN1. A recent review reported the prevalence of neoplasms as follows: primary hyperparathyroidism (PHPT), 95%; pituitary NETs, 30-40%; pancreatic NETs, 30-70%; and adrenocortical tumors, 40% (2). Typically, they show a mutation in the *MEN1* gene as genotype-positive (GP)-MEN, although 10-30% are genotype-negative (GN)-MEN (3).

Excess endocrine hormones induce many related complications such as osteoporosis due to parathyroid hormone or cortisol (4) and glucose intolerance due to cortisol (5), aldosterone (6) or growth hormone (7). These disorders are often asymptomatic and are difficult to diagnose (8). Therefore, clinical features such as a buffalo hump suggesting Cushing’s syndrome and an overgrown jaw or tongue suggesting acromegaly are clues for diagnosis (9). Headache is also a critical symptom suggesting a pituitary tumor (10). However, these features can be easily overlooked, resulting in a delayed diagnosis (11).

Herein, we report a case of GN-MEN1 diagnosed through the investigation of hyperglycemia and headache accompanied by a prolactinoma, PHPT, and subclinical Cushing’s syndrome. Immediate treatment of each endocrine disorder resulted in the withdrawal of insulin injections and oral hypoglycemic agents (OHAs). The present case highlights the importance of further investigation and appropriate interventions for endocrine disorders, even in patients with GN-MEN or sporadic cases.

## 2 Case report

A 56-year-old female was referred to our hospital for the management of hyperglycemia identified during a health check-up at her workplace. At first appearance at the outpatient clinic, she had hemoglobin A1c (HbA1c), 12.0%; fasting plasma glucose (FPG), 16.3 mmol/L (294 mg/dL); C-peptide, 1.8 nmol/L (5.35 ng/mL); and immunoreactive insulin (IRI), 457.0 pmol/L (63.7 μIU/mL), suggesting insulin resistance. Anti-glutamic acid decarboxylase autoantibody was negative [< 5.0 kU/L (U/mL)], and type 2 diabetes was suspected. A urine test revealed glucose (-), ketones (+/-), and proteins (+/-). Due to the risk of progression of ketosis, insulin therapy was immediately initiated (insulin Glargine 3U/day).

Additionally, she complained of headaches, and further medical interviews revealed a history of prolactinoma, diagnosed at 28 years of age, and kidney stones at 33 years of age. Her endocrinological family history included Graves’ disease (older brother) and pancreatic ductal cancer (paternal grandfather) without diabetes. Regarding the prolactinoma, the patient had been prescribed cabergoline before pregnancy. However, she stopped visiting the hospital and taking medications immediately after delivery at the age of 30. She sometimes had galactorrhea and headaches but did not visit the hospital. Based on this history, endocrine laboratory data was added that showed the prolactin was elevated to 1102.0 μg/L (ng/mL) with luteinizing hormone (LH) < 0.1 mIU/mL (IU/L) and follicular stimulating hormone (FSH) 2.7 mIU/mL (IU/L), as hyperprolactinemia and hypogonadism. The lipid profile was also impaired [triglyceride (TG), 4.8 mmol/L (429 mg/dL); low-density lipoprotein (LDL), 6.5 mmol/L (250 mg/dL)]. However, familial hypercholesterolemia was not established with Achilles tendon X-ray assessment (7.2 and 7.1 mm in right and left). She was then admitted to our hospital for the management of hyperglycemia with an examination of endocrine parameters.

**Figure 1:**
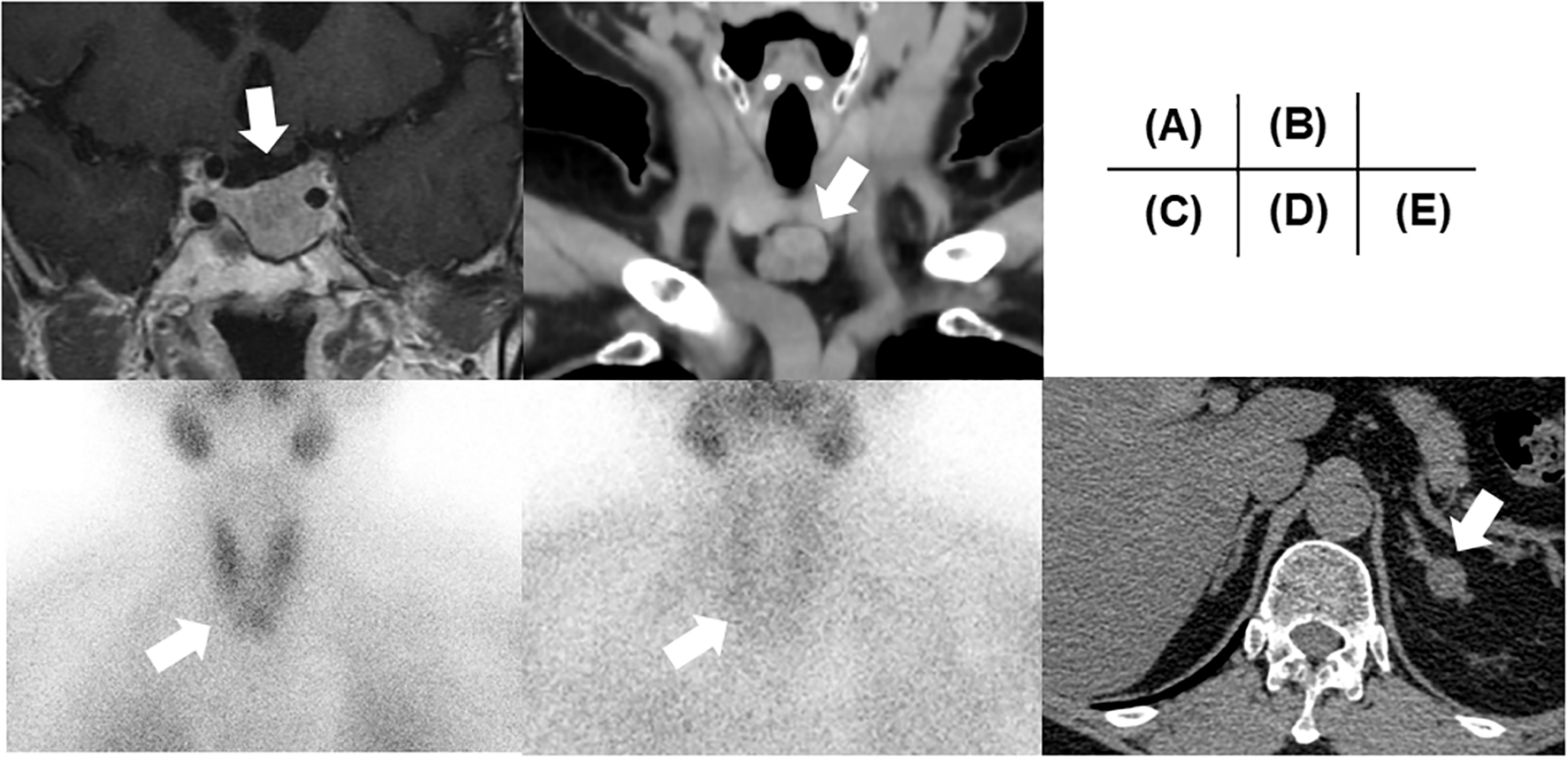
Clinical images. **(A)** A pituitary image on MRI. **(B)** A thyroid isthmus image on CT. **(C)** Early- and **(D)** late-phase of ^99m^Tc-MIBI. **(E)** The left adrenal tumor on CT. All tumors are highlighted with a white arrow in each image. MRI, Magnetic Resonance Imaging; CT, computed tomography; MIBI, methoxyisobutylisonitrile.

On admission, her height and body weight were 159.6 cm and 70.6 kg (body mass index [BMI] 27.7 kg/m^2^). Blood pressure and heart rate were 100/62 mmHg and 67 bpm, respectively. Body temperature was 36.6°C. Physical examination revealed no abnormalities and no galactorrhea was observed. She had not experienced decreased body weight or hyperphagia before admission. Her previous maximum body weight was 70.0 kg at 55 years of age, which was gradually increased after delivery (60 kg at 30 years of age) and not changed for recent several months. She works as a store clerk of the supermarket and living with her daughter with regular three meals a day without apparent diabetes-influencing habits including additional snacks. Her physical activity level was ordinary exertion as mainly static activity engaged in the seated position (12). Therefore, we proposed her to start diet therapy as 1700 kcal/day (30 kcal/kg of ideal body weight/day). The blood sample data at resting position showed reproducible high prolactin levels and hypogonadism (**Table 1**). Due to the absence of galactorrhea, and amenorrhea could only be derived from menopause, the presence of macroprolactinemia was investigated. Polyethylene glycol (PEG) precipitation, gel filtration chromatography and IgG isolation using a protein G column were performed, which indicated no macroprolactinemia; PEG precipitation ratio of prolactin was 45.8% (reference range; ≤ 57%), prolactin was eluted at the position of 23kDa monomeric prolactin, and protein G-bound prolactin ratio was 0.6% (reference rage; < 5%) (13). Magnetic resonance imaging revealed a 19 mm diameter pituitary tumor (Knosp grade 3) with downward progression (**Figure 1A**). Computed tomography (CT) revealed no pancreatic tumor. Based on these data, the patient was diagnosed with a prolactinoma without impaired pituitary or pancreatic hormone levels. Fortunately, the patient did not have diabetic retinopathy or temporal defects in a visual field test. Additionally, she showed normal-high calcium [2.50 mmol/L (10.0 mg/dL, reference range: 2.10–2.55 nmol/L)] with an elevated serum parathyroid hormone (PTH) level (intact PTH 84.0 pg/mL, reference range 10.0-65.0 pg/mL) on admission and this trend was reconfirmed as serum calcium 2.3 mmol/L (9.4 mg/dL) and intact PTH 118.0 pg/mL few months later. Additional ultrasonography revealed a thyroid isthmus tumor similarly detectable on CT (**Figure 1B**) with renal calcinosis. In the early phase, the tumor showed radiopharmaceutical accumulation of 99^mTc^-methoxyisobutylisonitrile, which also showed weak enhancement in the late phase (**Figures 1C, D**). We also measured 24-hour urine calcium level on two separate periods, which revealed to be hypercalciuria [6.96 and 13.24 mg/day (reference range: < 6.25 mmol/day (250 mg/day) (14)] and FECa was 0.027 (2.7%) [> 0.01(1%)], as negative suggestion for familial hypocalciuric hypercalcemia. Based on these data, the patient was additionally diagnosed with PHPT. Furthermore, a left adrenal tumor (16 mm in diameter) was observed on CT (**Figure 1E**). Her serum cortisol level after a 1 mg dexamethasone suppression test was 107.6 nmol/L (3.9 μg/dL) [> 49.7 (1.8 μg/dL]), suggesting autonomous cortisol secretion. ACTH was not suppressed at baseline [8.5 pmol/L (38.7 pg/mL) [> 2.2 pmol/L (10.0 pg/mL)] in the morning, and daily fluctuations of cortisol were maintained [113.1 nmol/L (4.1 µg/dL) [< 138.0 nmol/L (5.0 µg/dL) at night]. The tumor showed uptake of adosterol without contralateral suppression. Therefore, the patient was provisionally diagnosed with mild autonomous adrenal cortisol secretion (MACS) (15). There were no spine fractures on X-ray and T-scores of -2.5 (71% of the young adult mean) and -1.8 (75% of the young adult mean) for the lumbar spine and femoral neck, respectively, by dual-energy X-ray absorptiometry indicated osteoporosis (16). Integrated endocrine diseases, such as the coexistence of prolactinoma and PHPT (with an adrenal tumor) led to the diagnosis of MEN1. Her family history suggested that her MEN might be a sporadic onset.

**Table 1:** The laboratory data are shown. Endocrinological parameters of diurnal rhythm are measured at each timepoint after the resting position for 30 minutes.

<Diabetic data>		<Endocrinological data>	8:00 AM	11:00 PM
HbA1c (%)	12.0	ACTH (pmol/L)	8.5	3.8
FPG (mmol/L)	16.31	cortisol (nmol/L)	347.6	113.1
plasma insulin (pmol/L)	457.0	PRA (ng/mL/hr)	1.1	1.6
C-peptide (nmol/L)	1.8	PAC (ng/dL)	1.5	7.64
anti-GAD antibody (kU/L)	<5.0	prolactin (μg/L)	1084.6	1118.8
		LH (IU/mL)	<0.1	<0.1
<Lipid profile>		FSH (IU/mL)	1.4	1.5
TC (mmol/L)	9.1	GH (μg/L)	0.2	
LDL (mmol/L)	6.5	IGF-1 (nmol/L)	17.1	
TG ((mmol/L)	4.8	TSH (mIU/L)	0.9	
HDL ((mmol/L)	1.5	Free T3 (pmol/L)	3.8	
		Free T4 (pmol/L)	0.1	
<Urine data>		intact PTH (pg/mL)	84.0	
protein	(+/-)	DHEA-s (µmol/L)	5.4	
glucose	(-)	estradiol (pmol/L)	44.0	
blood	(-)	glucagon (ng/L)	19.3	
ketone	(+/-)	calcitonin (ng/L)	<0.5	
ACR (mg/mmoL⬝Cre)	4.2	25-hydroxy-vitamin D (nmol/L)	34.4	
<Blood chemistry data>				
total protein (g/L)	79.0	creatinine (μmol/L)	52.2	
albumin (g/L)	48.0	eGFR (mL/s)	1.3	
total bilirubin (g/L)	15.5	uric acid (μmol/L)	309.3	
AST (U/L)	61	Na (mmol/L)	137.0	
ALT (U/L)	105	K (mmol/L)	3.90	
γGTP (U/L)	95	Cl (mmol/L)	96.0	
ALP (U/L)	172	Ca (mmol/L)	2.50	
LDH (U/L)	203	IP (mmol/L)	1.32	
BUN (mmol/L)	4.9	CRP (mg/L)	2.7	

HbA1c, hemoglobin A1c; FPG, fasting plasma glucose; GAD, glutamic acid decarboxylase; TC, total cholesterol; LDL, low-density lipoprotein; TG, triglyceride; HDL, high-density lipoprotein; ACR, albumin-creatinine ratio; AST, aspartate aminotransferase; ALT, alanine aminotransferase; γGTP, gamma-glutamyltransferase; ALP, alkaline phosphatase; LDH, Lactate dehydrogenase; BUN, urea nitrogen; ACTH, adrenocorticotropic hormone; PRA, plasma renin activity; PAC, plasma aldosterone concentration; LH, luteinizing hormone; FSH, follicle- stimulating hormone; GH, growth hormone; IGF-1, insulin like growth factor-1; TSH, thyroid-stimulating hormone; PTH, parathyroid hormone; DHEA-s, dehydroepiandrosterone sulfate; eGFR, estimated glomerular filtration rate; CRP, C-reactive protein.

**Figure 2:**
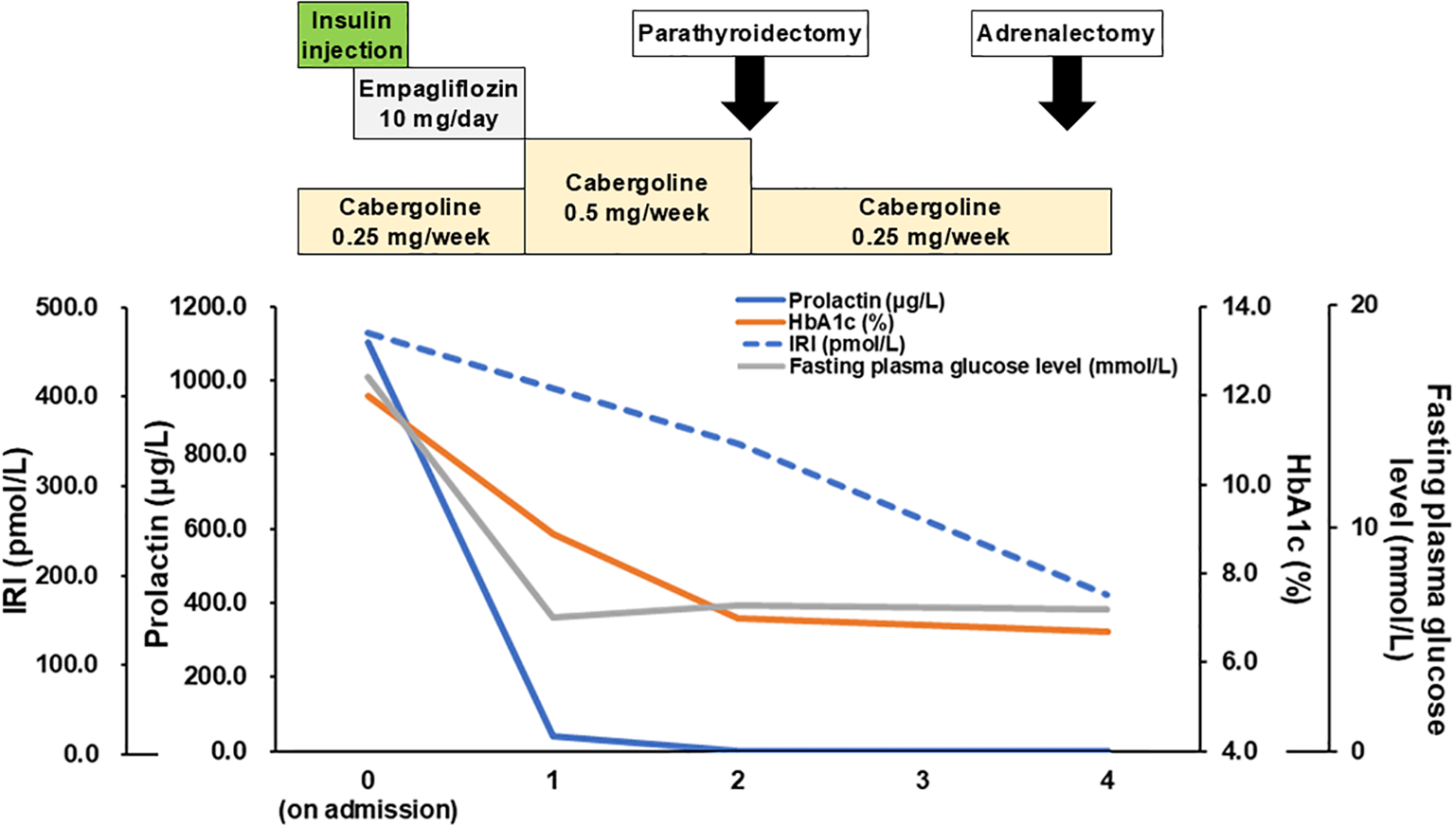
Clinical time-course. Time course of medication and laboratory data (prolactin, HbA1c, IRI, FPG) are shown. HbA1c, hemoglobin A1c; IRI, immunoreactive insulin; FPG, fasting plasma glucose.

For glucose management, basal-bolus insulin therapy was modulated. At first, we shifted to the basal-bolus therapy and started insulin Aspart 6-4-4 units and insulin Glargine 0-0-6-0 units from 1^st^ day of admission. The doses were increased to insulin Aspart 8-6-8 units and insulin Glargine 0-0-13 units per day at maximum. After resolving the glucose toxicity, an OHA, empagliflozin (10 mg), was administered at 8^th^ day of admission, and insulin therapy was gradually withdrawn resulting ceased at 15^th^ day of admission. A prolactin suppression test with bromocriptine (2.5 mg) showed a 66.0% decrease (1128.9 μg/L at baseline and 383.5 μg/L at six hours as the bottom), suggesting the effectiveness of dopamine agonists. Mainly for the management of controlling tumor growth (17), cabergoline 0.25 mg/week was initiated. Due to the risk of cerebrospinal fluid leakage, there has been careful follow-up contact with a neurosurgeon; no leakage has occurred, to date. After initiating cabergoline, the serum prolactin level decreased in parallel with the glucose level (prolactin (μg/L)/HbA1c (%)/FPG (mmol/L), 41.0/8.9/5.8 at one month; 2.1/7.0/6.4 at three months and 1.5/6.7/6.2 at four months after initiation of cabergoline), even though empagliflozin 10 mg was discontinued one month after cabergoline initiation. The patient has a well-controlled HbA1c level, to date. Moreover, her insulin resistance also improved with cabergoline treatment [IRI (pmol/L)/homeostasis model assessment of insulin resistance (HOMA-IR), 335.8/13.28 at two months and 171.5/6.60 at four months] (18). The clinical time course is shown in **Figure 2**.

**Figure 3:**
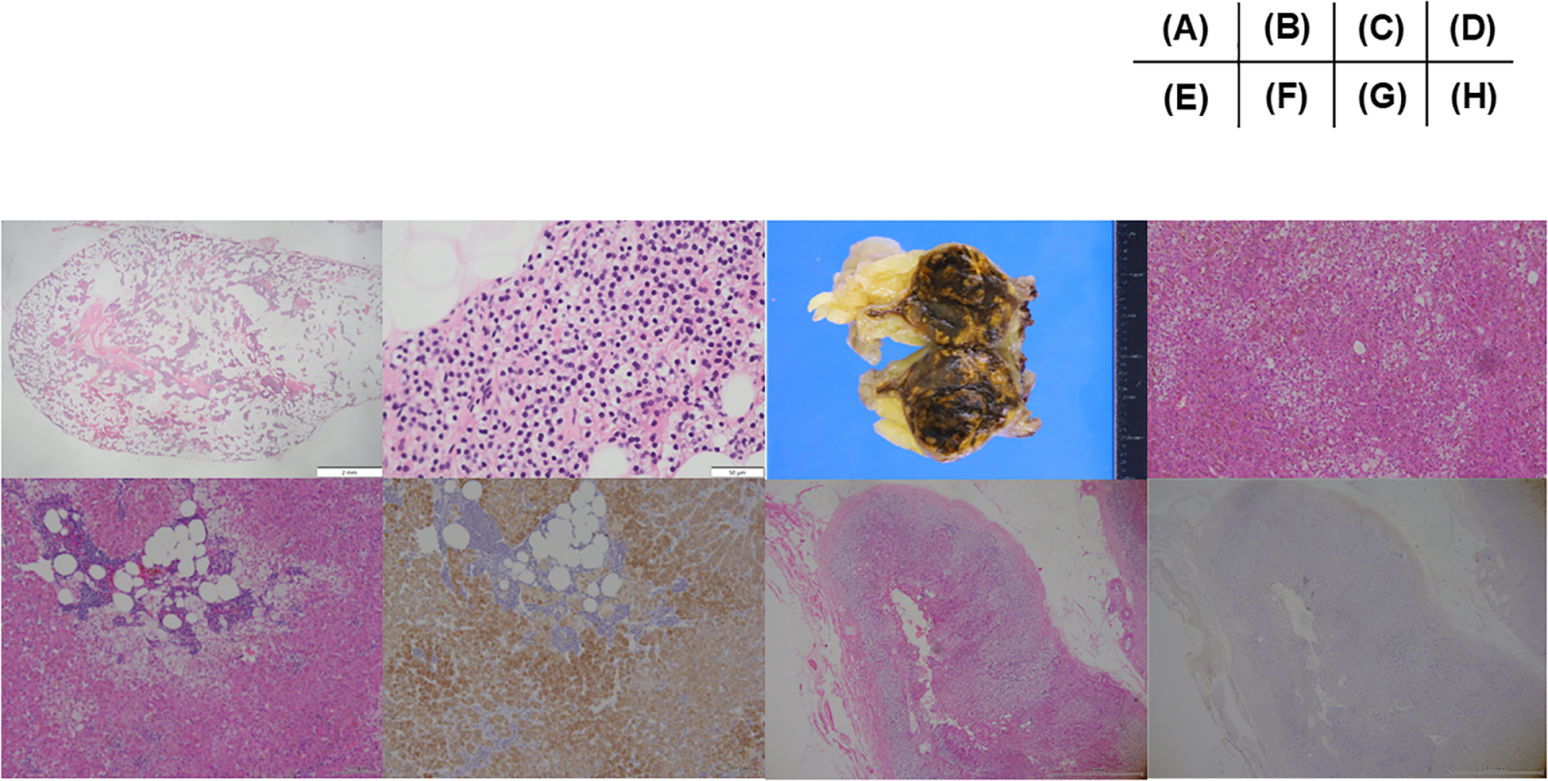
Pathological images. **(A)** A low-power and **(B)** high-power field image of parathyroid specimen removed as left lower parathyroid tumor. Histopathologically, dominant chief cells with oxyphil cells with relatively minimal changes of cytoplasmic fat within cells, consistent with parathyroid adenoma or hyperplasia. **(C)** A macroscopic image of the removed left adrenal gland. The tumor consists of dominant brown area with interspace yellow areas. **(D)** Histopathological features of the adrenocortical adenoma. Tumor cells harboring eosinophilic cytoplasm with lipofuscin were intermingled with those clear cytoplasm (Hematoxylin and eosin stain). **(E)** Histopathological features of adenoma: Lipomatous change with intratumoral lymphocytes infiltration was detected in the tumor tissue (Hematoxylin and eosin stain). **(F)** Immunohistochemistry of c17. Marked immunoreactivity of c17 in tumor cells in the same area as illustrated in **(E)**. **(G)** Histopathological features of non neoplastic attached adrenal gland. Moderate degree of cortical atrophy of zona fasciculata and reticularis was detected (Hematoxylin and eosin stain). **(H)** Immunohistochemistry of DHEA-ST in non neoplastic attached adrenal gland in the same area as **(G)**. DHEA-ST immunoreactivity was not detected in the zona reticularis, indicating the long-term suppression of hypothalamus-pituitary axis of the patient prior to surgery. All the specimen were diagnosed and confirmed by the board certificated pathologists.

Regarding PHPT, the patient fulfilled the criteria for surgery (evidence of nephrocalcinosis or nephrolithiasis and hypercalciuria) (14, 19) and the operation for removing a tumor at the thyroid isthmus was performed two months after initiation of cabergoline. During operation, thyroid isthmus tumor with adjacent structures of muscle and adipose tissue was confirmed. Surgeons also recognized pea-sized tumor at lower left of the thyroid attached to the thyroid isthmus tumor we could not confirmed at other modalities, suggesting parathyroid gland. No other enlargement of parathyroid gland was confirmed during operation. Therefore, the thyroid isthmus tumor and left lower parathyroid-like tumor are resected with these adhesive tissues. The thyroid isthmus tumor was revealed to be multiple papillary carcinoma tumors (pT3 N0 M0 with strap muscles and adipose tissue invasion) containing adenomatous goiter. The pea-sized parathyroid tumor was confirmed as the parathyroid gland, pathologically suggesting hyperplasia or adenoma (**Figures 3A, B**). Intact PTH was normalized 5 months after operation [intact PTH 43.0 pg/mL and serum calcium 2.44 mmol/L (9.8 mg/dL)]. Otherwise about MACS, females under 65 years of age are reported to have higher all-cause mortality (20) and left adrenalectomy was performed at four months after initiation of cabergoline. Histopathologically, an adrenal adenoma is confirmed as the cortisol producing adrenocortical adenoma positive in steroidogenic enzymes with lipomatous changes and intratumoral lymphocytes infiltration (21) (**Figures 3C–F**). Weiss score was 1 point (≤ 3, diagnosed as benign), positive for “clear cells ≤ 25% of the tumor volume. Additionally, moderate atrophy and absence of dehydroepiandrosterone sulfotransferase (DHEA-ST) immunoreactivity at non neoplastic adrenal gland, suggesting long-term suppression of hypothalamus-pituitary axis, was confirmed. Therefore, the diagnosis was changed to subclinical Cushing’s syndrome from MACS (22), suggesting autonomous cortisol secretion (**Figures 3G, H**). After operation, her morning cortisol was slightly decreased as 262.1 nmol/L (9.5 μg/dL). The lipid profile improved to TG 1.8 mmol/L (162 mg/dL) and LDL 2.2 mmol/L (86 mg/dL) with rosuvastatin 5 mg.

After informed consent was obtained, target gene testing was performed for *MEN1* and mimicking the disease. Gene panel analysis was performed at the Kazusa DNA Research Institute (Kisarazu, Chiba, Japan) using next-generation sequencing. The target genes were exons of *MEN1, CDKN1B, RET, CASR, GNA11, AP2S1, CDC73 and GCM2*. Genomic DNA was extracted from the leukocytes in the peripheral blood. A synonymous variant of the *MEN1* gene was found in c.1254C>T (D418D, rs2071313), which is a common polymorphism that is thought to be non-pathogenic (23, 24).

Additional written informed consent was obtained from the patient for the publication of the clinical details and images. The requirement for formal ethical approval was waived because this is a case report at Ohta Nishinouchi Hospital.

## 3 Discussion

This report describes a case of GN-MEN1 accompanied by prolactinoma, PHPT, and MACS. In this case, hyperglycemia and headache led to the diagnosis of an endocrine disorder. MEN1-associated manifestations may have concomitantly affected the metabolic disorders, including diabetes.

Regarding the MEN1 genotype, the present case did not show any known pathogenic variants in the *MEN1* exons. GN-MEN1 has been reported to exist in 10-30% of clinically diagnosed cases of MEN1 (3). GN-MEN1 has a different clinical course than GP-MEN1. For instance, GN-MEN1 often does not develop into GEP-NET and has a single-gland PHPT compared to GP-MEN1 (25). GN-MEN1 patients are also known to lack a family history of MEN1 (26) which are consistent with the present case. Intriguingly, MEN1 subjects with PHPT can remain asymptomatic for a long period and mild-moderate hypercalcemia with increased PTH could be shown typically, like our case (27, 28). This period is thought to be derived from the duration that the elevated PTH can consume the 25-hydroxy-vitamin D. These mechanisms might affect the normal-high calcium levels and decreased 25-hydroxy-vitamin D is also confirmed in our case. GN-MEN1 has been reported to show a favorable clinical course with a decreased prevalence of MEN1-associated tertiary manifestations (3), which is inconsistent with the present case. Notably, the delayed diagnosis of MEN1-associated tumors, particularly prolactinoma, could have been crucial in this case. Invasive papillary thyroid carcinoma (PTC) was also confirmed and crucial in the present case. The potential association between MEN1 and PTC has been proposed in some reports (29, 30), although which is still controversial. Furthermore, the evidence of association between GN-MEN1 and PTC was not found as far as we searched and this could happen by chance. These associations also need further research. To summarize, precise investigations of many endocrine neoplasms are required even for sporadic or GN-MEN1 cases.

Another cautionary point is that the target gene sequence could partly vary by introducing novel sequencing techniques and deletion/duplication analyses. The intronic variants could not be assessed for this case. A previous report revealed that the intronic splicing site variant associated with MEN1 was accompanied by a prolactinoma, PHPT, and cortisol-producing adrenal adenoma, as in the present case (31). The whole-genome sequence, including the intron site, may reveal further genotype-phenotype correlations in MEN1. These questions require further investigation.

The diagnosis of MEN1 itself could affect glucose metabolism, as reported in patients with MEN1, who have an increased prevalence of diabetes mellitus and impaired fasting glucose level, even among carriers of a *MEN1* gene mutation (32, 33). Uncontrolled hypercalcemia and evidence of GEP-hyperstimulation are associated with glucose intolerance (32). Thus, MEN1 can be one of the differential diagnoses as the cause of diabetes. Additionally, diabetes is well-known to promote atherosclerosis and increase mortality via cardio-cerebrovascular disease (34, 35). Although the known prognostic factors for MEN1 are thymic or GEP-NETs (36), a recent report showed that the most frequent non-endocrine-related cause of death in patients with MEN1 is coronary disease, followed by non-MEN1-related neoplasms and cerebrovascular disease (37). Therefore, glucose management in patients with MEN1 is vital to their prognosis.

MEN1-associated hormones also induce glucose intolerance. Prolactin and prolactinomas are known to induce glucose intolerance via increased insulin resistance, resulting in β cell dysfunction (38), increased BMI, and an impaired lipid profile (39). Therefore, the transition in prolactin level could reflect the time course of treatment in the present case. In addition, cabergoline independently affected the glucose level. Cabergoline has been reported to increase dopamine and decrease the noradrenergic level in the hypothalamus, resulting in improved insulin sensitivity and reduced liver gluconeogenesis, even in patients with type 2 diabetes, and it is expected to be a new therapeutic drug for diabetes (40, 41). PHPT, an excess parathyroid hormone, has also been reported to increase the prevalence of diabetes via increased insulin resistance (42). Similarly, excess cortisol, such as MACS or subclinical Cushing’s syndrome, results in impaired insulin secretion and resistance (43). Although the possibility of false-positive in 1mg DST exists because of physical and mental stress or sleep apnea syndrome via elevation of cortisol level (44, 45), atrophy of the attached adrenal cortex and suppression of DHEA-ST immunoreactivity supports cortisol excess from the tumor. However, considering the time-course of the treatment shown in **Figure 2**, the effect of PHPT and cortisol producing adenoma might be minimal.

In summary, the combination of manifested endocrine disorders in the present case affected the glucose level. Significantly, the prolactin level was elevated. Therefore, normalization of the prolactin level may improve the metabolic profile in postmenopausal females, although the significance of the treatment of prolactinoma in postmenopausal females remains controversial. As a limitation, the resolution of glucotoxicity with insulin or parathyroidectomy also affected continuous improvement in the glucose level, in the present case.

Collectively a case of prolactinoma diagnosed as GN-MEN1 through the investigation of hyperglycemia has been described. The possibility of endocrine disorders, including MEN1 should be considered, at the same time, particularly in patients with suspicious symptoms or MEN1-related neoplasms with hyperglycemia, even in sporadic or GN-MEN.

## Data Availability

The datasets presented in this article are not readily available because of ethical and privacy restrictions. Requests to access the datasets should be directed to the corresponding author/s.
